# Fadrozole-mediated sex reversal in the embryonic chicken gonad involves a PAX2 positive undifferentiated supporting cell state

**DOI:** 10.3389/fcell.2022.1042759

**Published:** 2022-11-10

**Authors:** Martin A. Estermann, Craig A. Smith

**Affiliations:** Department of Anatomy and Developmental Biology, Monash Biomedicine Discovery Institute, Monash University, Melbourne, VIC, Australia

**Keywords:** fadrozole, PAX2, gonad, sex-reversal, estrogen, chicken

## Abstract

Gonadal sex differentiation among vertebrates involves divergent fates of a common group of progenitor cells present in both presumptive ovaries and testes. The first cell type to differentiate gives rise to pre-Sertoli cells in the testis, and pre-follicular cells in the ovary. These cells derive from a common lineage of so-called “supporting cells”. In birds and other egg-laying vertebrates, locally synthesised estrogen has a central role in ovarian development and influences the fate of these supporting cells. Manipulation of estrogen levels during embryonic development induces gonadal sex reversal, providing an experimental setting to evaluate the process of gonadal sex differentiation. Recently, we identified PAX2 as a novel marker of the undifferentiated supporting cell lineage in the chicken embryo, expressed in both sexes prior to overt gonadal sex differentiation. *PAX2* expression is downregulated at the onset of gonadal sex differentiation in both males and females. The analysis of this undifferentiated supporting cell marker, together with Sertoli (male) and pre-granulosa (female) will enhance our understanding of supporting cell differentiation. Here we characterized the supporting cells differentiation process and identified undifferentiated supporting cells in estrogen-mediated sex reversal experiments. Female embryos treated with the aromatase inhibitor fadrozole developed into ovotestis, containing pre-granulosa cells, Sertoli cells and PAX2 positive undifferentiated supporting cells. In contrast, male embryos treated with 17β-estradiol showed no PAX2^+^ undifferentiated gonadal supporting cells. Fadrozole time-course as well as multiple dose analysis suggests that supporting cell transdifferentiation involves a dedifferentiation event into a PAX2^+^ undifferentiated supporting cell state, followed by a redifferentiation towards the opposite sex lineage.

## Introduction

Gonadal sex differentiation describes the process of ovary or testis formation among vertebrate embryos. In most species, the gonads differentiate during embryonic or larval life, or shortly thereafter. Among humans, gonadal and external genital differentiation are sometimes atypical. Differences of Sex Development (DSDs) in humans have an incidence of around 1% of all live births. DSDs occurs when the chromosomal, gonadal or anatomical sex are discordant or ambiguous (Vilain, 2011; Eggers et al., 2016). Gonads exhibit a wide range of phenotypes, from total to partial sex reversal to gonadal dysgenesis (Gomes et al., 2020). Despite the advances genetic diagnosis, many DSD cases lack a definitive molecular diagnosis (Estermann and Smith, 2020). All current DSDs diagnostical methods do not provide functional information. This is crucial for understanding the etiology and risks associated with specific DSDs mutations, and for clinical management (Barseghyan et al., 2018). In recent years, animal models have been very instructive in elucidating the molecular genetics of typical and atypical gonadal sex differentiation (Sutton et al., 2011; Croft et al., 2018; Gonen et al., 2018; Robevska et al., 2018; Garcia-Acero et al., 2020).

Comparative analysis of chicken and mouse embryos has demonstrated significant genetic and morphological conservation of gonadal sex differentiation (Ayers et al., 2013a; Ayers et al., 2015). As in human embryos, both chicken and mouse exhibit the same groups of progenitor cell types in the gonad. These are the supporting cells (presumptive Sertoli cells in the testis and granulosa cells in the ovary), purely steroidogenic cells (presumptive Leydig and theca cells), germ cells (sperm or ova) and other interstitial cells such as vascular progenitors (Estermann et al., 2020; Garcia-Alonso et al., 2022; Mayere et al., 2022). This conservation largely extends to the genetic level. *DMRT1*, *AMH,* and *SOX9* are expressed and play decisive roles in testicular morphogenesis, while *WNT4*, *RSPO1*, *FOXL2,* and *CYP19A1* (aromatase) are important in the ovary (Smith and Sinclair, 2004; Ayers et al., 2013b; Cutting et al., 2013; Lambeth et al., 2014). Therefore, the chicken provides a very tractable model for studying embryonic gonad development (Morrish and Sinclair, 2002; DeFalco and Capel, 2009). In the chicken embryo, gonad formation begins at embryonic day 3.5 (E3.5), Hamburger and Hamilton developmental stage 19 (HH19) (Hamburger and Hamilton, 1951). At this stage, gonads are visible in the ventromedial surface of the mesonephric (embryonic) kidneys. During these early stages the gonads are histologically and transcriptionally similar between the sexes (bipotential/undifferentiated) (Ayers et al., 2015; Estermann et al., 2020). Sex differentiation in birds commences around E6-6.5 stating the differentiation of the supporting cells into Sertoli cells in ZZ males and pre-granulosa cells in ZW females (Ayers et al., 2015; Estermann et al., 2020). By E9.5 male and female gonads are morphologically and histologically completely different. Seminiferous cords form in the medulla, containing both Sertoli and germ cells. In females, the left ovary develops a thick cortex where the germ cells are located, while the right one regresses. Additionally, the medulla forms vacuolated structures called lacunae. By E12.5 the supporting cell lineage is already properly differentiated, whereas the germ line commences to sexually differentiate (Smith et al., 2008).

In birds and other egg-laying vertebrates, locally synthesised estrogen is required for ovarian development (Smith et al., 1997; Pieau and Dorizzi, 2004; Brunstrom et al., 2009; Barske and Capel, 2010; Guiguen et al., 2010). The estrogen synthesising enzyme, aromatase, is only expressed in female gonads at the onset of ovarian differentiation (Elbrecht and Smith, 1992; Smith et al., 1997). The gene encoding aromatase, *CYP19A1*, becomes activated in cells of the inner medulla of female embryos from embryonic day 6.0 (stage 29/30). Experimental manipulation of estrogen levels during embryonic development has been associated with gonadal sex reversal (Guioli et al., 2020; Shioda et al., 2021). Inhibition of aromatase enzyme with the drug fadrozole, leads to testis or ovotestis formation in genetic females (Elbrecht and Smith, 1992; Vaillant et al., 2001a; Vaillant et al., 2001b; Smith et al., 2003). Meanwhile, while aromatase over-expression in genetic males can induce transient ovary formation (Lambeth et al., 2016). The masculinizing effects of fadrozole depend upon the time and stage of exposure. When the drug is injected into eggs between E3.5- E4 (developmental stage 19–21), blockade of estrogen action and extensive masculinization is seen. When injected after the onset of gonadal sex differentiation at E6.0 (stage 29), the effects of aromatase inhibition are less marked. That is, aromatase must be inhibited in genetically female embryos before it starts catalyzing estrogen at E6.0 (Abinawanto et al., 1997; Etches et al., 1997; Vaillant et al., 2003). This model provides an experimental setting to evaluate the process of gonadal sex reversal and gonadal sex differentiation more broadly.

Recently, we identified PAX2 as a novel marker of the undifferentiated supporting cell population in the embryonic chicken gonad. Furthermore, we found that PAX2 is also expressed in the undifferentiated gonads of all major avian lineages (Estermann et al., 2021a). In chicken, PAX2 expression is sharply downregulated at the onset of gonadal sex determination in both males and females (Estermann et al., 2020; Estermann et al., 2021a). The identification of this undifferentiated supporting cell marker, together with Sertoli (male) and pre-granulosa (female) markers now allows for a more complete understanding of the timing and nature of supporting cell differentiation.

Here we characterize the supporting cells differentiation process, and the existence of undifferentiated supporting cells in estrogen-mediated sex reversal experiments. Female embryos treated with the aromatase inhibitor, fadrozole, developed ovotestis, containing pre-granulosa cells, Sertoli cells and PAX2 positive undifferentiated supporting cells. In contrast, male embryos treated with 17β-estradiol showed no PAX2^+^ undifferentiated gonadal supporting cells. Fadrozole time-course as well as multiple dose analysis suggests that supporting cell transdifferentiation involves a dedifferentiation phase into a PAX2^+^ undifferentiated supporting cell state, followed by a redifferentiation towards the male (Sertoli) lineage.

## Materials and methods

### Chicken eggs

Fertilized *Gallus gallus domesticus* HyLine Brown eggs were obtained from Research Poultry Farm (Victoria, Australia) and incubated at 37.5°C under humid conditions. Embryos were staged according to Hamburger and Hamilton (Hamburger and Hamilton, 1951). Experiments were performed in accordance with our institutional animal ethics requirements (Approved Monash University AEC #17643).

### Fadrozole treatment

Eggs were injected at E3.5 (Hamburger and Hamilton stage HH19) (Hamburger and Hamilton, 1951) with 1 mg of fadrozole (Novartis) in 100 µl of PBS or vehicle, as described previously (Estermann et al., 2021b). Embryonic urogenital systems were collected at E6.5 (HH30), E9.5 (HH35) or E12.5 (HH38). For double injection experiments, eggs were injected at E3.5 and at E6.5 with 1 mg fadrozole in 100 µl of PBS or vehicle. Tissues were collected at E9.5.

### 17β-estradiol treatment

Eggs were injected at E3.5 (HH19) with 0.1 mg 17β-estradiol in 10% ethanol in sesame oil or vehicle, as described previously (Estermann et al., 2021b). Tissues were collected at E9.5 (HH35).

### Sexing PCR

Genetic sexing was performed by PCR, as described previously (Clinton et al., 2001). Genetic females (chromosomally ZW) were identified by the presence of a female-specific (W-linked) Xho I repeat sequence in addition to a 18S ribosomal gene internal control. Genetic males (chromosomally ZZ) showed the 18S band only (Clinton et al., 2001).

### Quantitative RT-PCR

Quantitative RT-PCR (qRT-PCR) was performed as reported before (Estermann et al., 2021a). Briefly, E9.5 (HH35) left gonads were collected and snap frozen in dry ice. After PCR sexing, three same-sex gonads from the same treatment were pooled for each sample, homogenized and RNA extracted using 1 ml TRIzol as per the manufacturer’s instructions, (TRIzol, ThermoFisher). Genomic DNA was removed using DNA-free™ DNA Removal Kit (Invitrogen) and 1 μg of total RNA was reversed transcribed into cDNA using Promega Reverse Transcription System (A3500). qRT-PCR was performed using the QuantiNova SYBR^®^ Green PCR Kit. *PAX2* expression levels were quantified by the 2^−ΔΔCT^ method using β-actin as internal control. PCR primers were: *PAX2* Fw: GGC​GAG​AAG​AGG​AAA​CGT​GA, *PAX2* Rv: GAA​GGT​GCT​TCC​GCA​AAC​TG, *β-Actin* Fw: CTC​TGA​CTG​ACC​GCG​TTA​CT and *β-Actin* Rv: TAC​CAA​CCA​TCA​CAC​CCT​GAT. Data was analysed using two-way ANOVA. Significance was determined using Tukey post-test.

### Immunofluorescence

Immunofluorescence of frozen sections was performed as reported previously (Estermann et al., 2020). Briefly, gonadal samples were fixed in 4% paraformaldehyde/PBS for 15 min, cryoprotected in 30% sucrose in PBS overnight, embedded in OCT embedding compound and snap frozen at −80°C. 10μm gonadal cryosections were permeabilized with 1% Triton X-100 in 1X PBS for 10 min, blocked in 2% BSA in 1X PBS for 1 h, incubated overnight at 4°C with the primary antibody in 1% BSA in 1X PBS. Primary antibodies used: rabbit anti-PAX2 (Biolegend 901001, 1; 500), rabbit anti-DMRT1 (in house antibody; 1:2,000), rabbit anti-SOX9 (Millipore AB5535, 1:4,000), rabbit anti-Aromatase (in house antibody; 1:4,000) and rabbit anti-AMH (Abexa ABX132175; 1:1,000). Samples were washed and incubated with the secondary antibody Alexa Fluor 488 donkey anti-rabbit (1:1,000) in 1% BSA in 1X PBS. Samples were counterstained with DAPI and mounted in Fluorsave (Millipore). Images were collected on a Zeiss Axio Imager A1 microscope using a Zeiss Axiocam MRc5 camera, using the same exposure time between treatments for expression comparisons. For PAX2 immunostaining, antigen retrieval was performed using the Dako PT Link automated system.

## Results

### PAX2 expression is induced upon fadrozole mediated masculinization at E9.5

**FIGURE 1:**
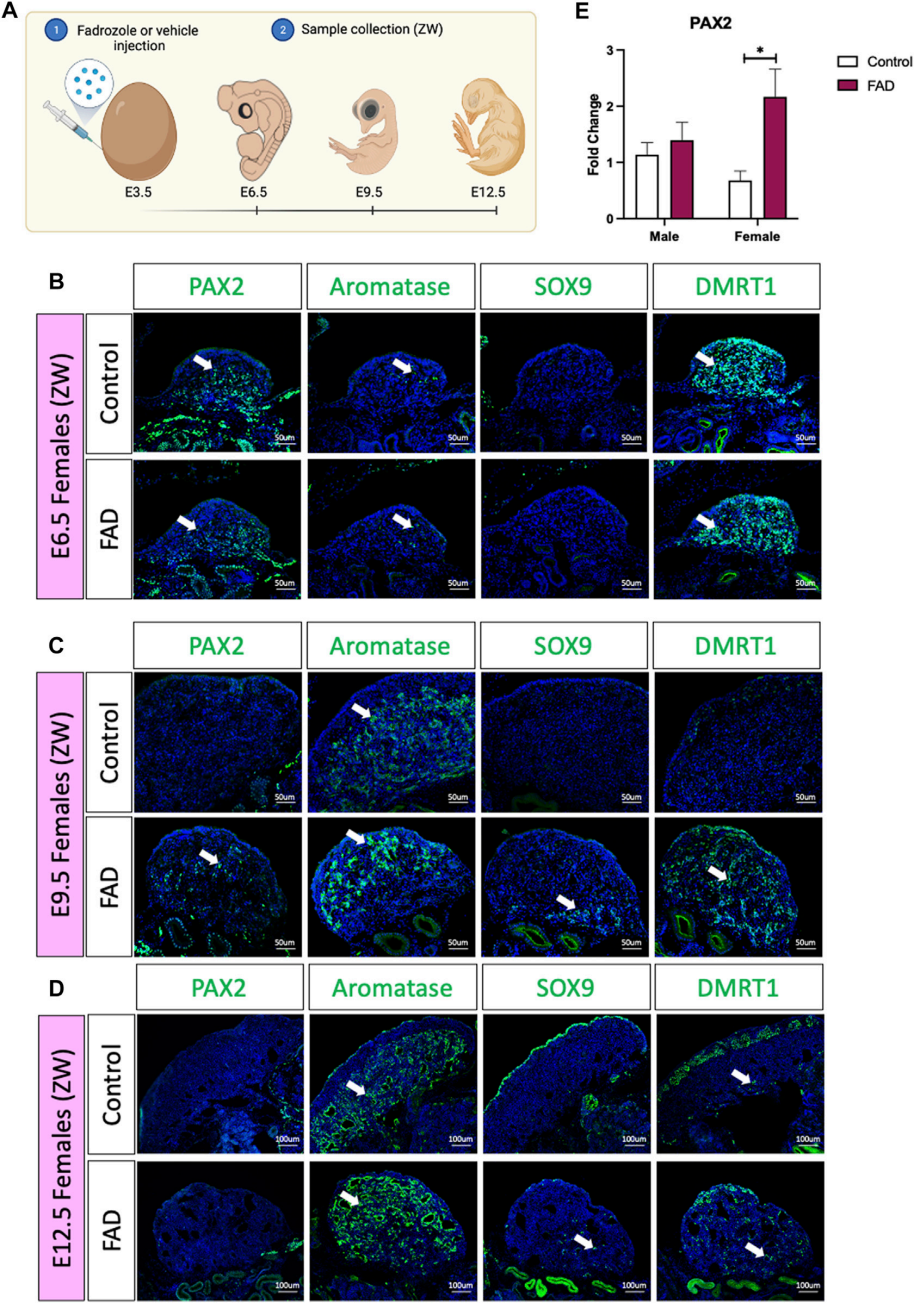
PAX2 expression is induced upon fadrozole mediated masculinization. **(A)** Schematic figure of the experimental plan. Fadrozole or vehicle solution was injected in chicken eggs at E3.5. Samples were collected at E6.5, E9.5, and E12.5 Immunofluorescence against PAX2, aromatase, SOX9 and DMRT1 in **(B)** E6.5, **(C)** E9.5 and **(D)** E12.5 female (ZW) gonads treated with fadrozole (FAD) or vehicle solution (Control). White arrows indicate positive cells. **(E)** *PAX2* qRT-PCR was performed in E9.5 gonadal samples of fadrozole (FAD) or vehicle (Control) treated embryos. Expression level is relative to β-actin and normalized to the male control. Bars represent mean ± s.e.m., *n* = 6. * adjusted *p* < 0.05. 2-way ANOVA and Tukey’s post-test.

In the chicken embryos, a typical genetic female develops a large ovary, characterised by thickened outer cortex and vacuolated underlying medulla (Vaillant et al., 2001a; Vaillant et al., 2001b). Treatment with a single dose of the aromatase inhibitor fadrozole results in gonads lacking a cortex and developing testicular cords rather than lacuna in the medulla (Vaillant et al., 2001b). To evaluate if fadrozole treatment in ZW embryos resulted in an increase of undifferentiated supporting cells throughout gonadal development, fadrozole was injected in E3.5 eggs and gonads were collected at E6.5, E9.5, and E12.5 (Figure 1A). Samples were genetically sexed and ZW gonads were immunostained for male (SOX9 and DMRT1), female (aromatase) and undifferentiated (PAX2) supporting cell markers. At E6.5, PAX2 was detected in both control and FAD-treated embryos (Figure 1B). Some numbers of aromatase^+^ cells were also detected, indicating the onset of ovarian differentiation (Figure 1B). At E9.5, control left ovaries were larger, expressing aromatase in the medulla but no PAX2, SOX9 or DMRT1 (Figure 1C). In contrast, fadrozole-treated female left gonads lacked a cortical compartment, and both male (SOX9) and female (aromatase) positive supporting cells co-existed in the same gonad, but in separated defined regions (Figure 1C). Aromatase positive cells were located in the apical region of the gonad whereas SOX9 positive cells were detected more basally, adjacent to the mesonephric kidney. Testis-associate DMRT1 was up-regulated throughout the medulla. PAX2^+^ cells were identified in the gonadal medulla, between SOX9 and aromatase positive supporting cells (Figure 1C). By E12.5, PAX2 expression had been extinguished suggesting that by this developmental stage, all the supporting cells committed to a pre-granulosa or Sertoli cell fate (Figure 1D). Control gonads showed a typical ovarian structure, with aromatase^+^ vacuolated medulla and an enlarged cortex, containing DMRT1 positive germ cells (Figure 1D). As expected, E12.5 fadrozole treated gonads lacked a properly defined cortex (Figure 1D). Aromatase positive pre-granulosa cells located extensively throughout the gonad, whereas SOX9^+^ DMRT1^+^, Sertoli-like cells, were in the basal region of the gonad (Figure 1D). This data suggests that by E12.5, the majority of the supporting cells in the medulla of fadrozole treated gonads at E3.5 differentiated into pre-granulosa cells.

To quantify the changes in PAX2 expression at E9.5, embryos were treated with fadrozole at E3.5, gonads were collected at E9.5 (Figure 1E), genotypically sexed and *PAX2* gonadal mRNA expression was measured by qRT-PCR. *PAX2* mRNA levels were significantly higher in female gonads (ZW) treated with fadrozole (FAD), compared with the vehicle control (Figure 1E). In males (ZZ), *PAX2* expression levels remained unchanged upon treatment (Figure 1E). These results indicate that fadrozole mediated masculinization results in an increase in gonadal PAX2^+^ expression, at E9.5, among cells of the female gonad. Based on our previous data, showing PAX2 to be a marker of prior to differentiation, these cells are interpreted to be undifferentiated supporting cells.

### PAX2 *expression is not induced upon estrogen-induced feminization*

**FIGURE 2:**
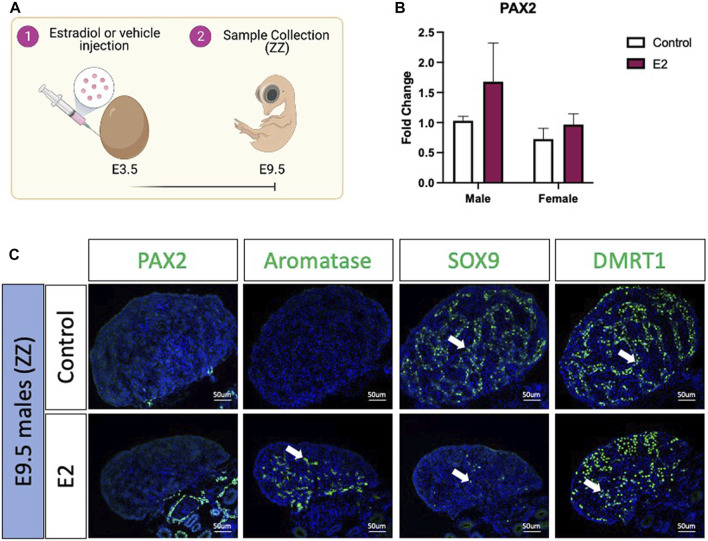
PAX2 expression is not induced upon estrogen-induced feminization. **(A)** Schematic figure of the experimental plan. 17β-estradiol or vehicle solution was injected in chicken eggs at E3.5. Samples were collected at E9.5. **(B)** *PAX2* qRT-PCR was performed in E9.5 gonadal samples of 17β-estradiol (E2) or vehicle (Control) treated embryos. Expression level is relative to β-actin and normalized to the male control. Bars represent mean ± s.e.m., *n* = 6. 2-way ANOVA and Tukey’s post-test. **(C)** Immunofluorescence against PAX2, aromatase, SOX9 and DMRT1 in E9.5 male (ZZ) gonads treated with 17β-estradiol (E2) or vehicle solution (Control). White arrows indicate positive cells.

To evaluate if PAX2 positive undifferentiated cells were also present in estradiol-mediated male to female sex reversed gonads, E3.5 chicken eggs were injected with 17β-estradiol (E2) or vehicle solution (Figure 2A). Embryos were collected at E9.5 (Figure 2A), genotypically sexed and *PAX2* expression was measured by qRT-PCR. No significant differences were found in *PAX2* mRNA expression levels, between treated (E2) and control gonads in both sexes (Figure 2B). PAX2 Immunofluorescence was performed on E9.5 male (ZZ) gonads treated with 17β-estradiol or vehicle, but no PAX2 positive cells were detected in the gonadal medulla (Figure 2C), consistent with the qRT-PCR results. To evaluate if, in fact, the gonads were sex reversed, aromatase, SOX9 and DMRT1 protein expression were evaluated by immunofluorescence. As expected, control male gonads showed no aromatase expression and medullary expression of SOX9 and DMRT1 (Figure 2C). In contrast, E2 treated gonads showed ectopic activation of aromatase and a reduced number of SOX9 and DMRT1 positive cells in the medulla (Figure 2C). Additionally, DMRT1 positive germ cells were detected in the cortical region of the gonad, as is seen in typical left ovaries. These results indicate that 17β-estradiol mediated sex reversal did not induce PAX2 positive undifferentiated supporting cells at E9.5.

### PAX2 undifferentiated supporting cells are lost upon booster injection of fadrozole

**FIGURE 3:**
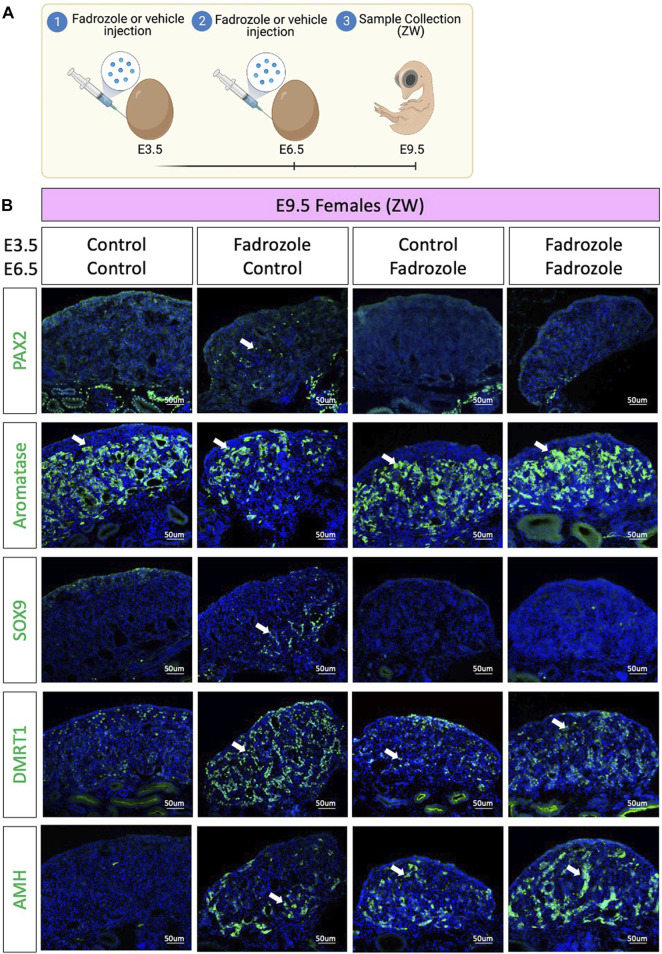
PAX2 undifferentiated supporting cells are lost upon booster injection of fadrozole. **(A)** Schematic figure of the experimental plan. Fadrozole or vehicle solution was injected in chicken eggs at E3.5 and E6.5. Samples were collected at E9.5. **(B)** Immunofluorescence against PAX2, aromatase, SOX9 and DMRT1 and AMH in E9.5 female (ZW) gonads treated with fadrozole or vehicle solution (Control). White arrows indicate positive cells.

As aromatase inhibition *via* fadrozole treatment at E3.5 only results in changes in PAX2 expression pattern at E9.5, but not in E6.5 or E12.5, we asked if those PAX2 undifferentiated cells were a consequence of peak fadrozole levels that then decay over time. To address this question, a double dose experiment was performed, where eggs were injected at E3.5 and E6.5 with vehicle solution (control) or fadrozole, generating four different conditions (Figure 3A). Embryos were collected at E9.5 and immunofluorescence for AMH, SOX9, DMRT1, PAX2 and aromatase was performed (Figure 3B). As expected, control ovaries expressed aromatase but no PAX2, SOX9, DMRT1 or AMH. Similar to the results from Figure 1C single dose of fadrozole at E3.5 resulted in gonads expressing male supporting markers (DMRT1, SOX9, AMH), female markers (aromatase) and undifferentiated PAX2 positive cells (Figure 3B). In contrast, single dose of fadrozole at E6.5 resulted in no PAX2 expression in the gonads, but both Sertoli-like and pre-granulosa cells coexisting in the gonadal medulla (Figure 3B). These results indicate that PAX2 upregulation is not a direct result of fadrozole treatment, and that the timing of fadrozole administration is important. Moreover, a similar phenotype was seen in gonads treated with double doses of fadrozole at E3.5 and E6.5 (Figure 3B). Despite both male and female markers coexisted in the gonadal mesenchyme, no PAX2 expression was detected (Figure 3B). This suggests that despite one dose at E3.5 results in PAX2 expression, a booster dose at E6.5 inhibits PAX2 expression at E9.5. Taking all together, these results suggests that the undifferentiated supporting cells present at E9.5 are not a direct result of fadrozole injection. Instead, this PAX2 undifferentiated population could be a result of the decay or metabolism of fadrozole at E9.5. The lack of PAX2 positive cells in the gonadal medulla when a booster dose of fadrozole is injected E6.5 supports this idea. The lack of functional fadrozole at E9.5 results in disinhibition of aromatase and the production of estrogen. Estrogen then could induce the differentiation into pre-granulosa cells while inhibiting Sertoli differentiation. The upregulation of PAX2 suggests that Sertoli to pre-granulosa trans-differentiation involves a dedifferentiation into undifferentiated PAX2^+^ supporting cells, followed by a redifferentiation towards pre-granulosa cells.

## Discussion

Gonadal sex reversal occurs when there is a discordance between the genetic/chromosomic and gonadal sex (Du et al., 2014). Despite the genetic sex is determined at fertilization, the gonads are sexually determined later during embryonic development (Wilhelm et al., 2007; Estermann and Smith, 2020). Initially, both male and female gonads develop similarly (Nef et al., 2019). These undifferentiated gonads have the potential of becoming a testis or an ovary, depending on the genetic or environmental signals they receive (Nagahama et al., 2021). Among the genetic signals, sex chromosome linked genes are sufficient and necessary for testicular development (Jager et al., 1990; Koopman, 1999; Smith et al., 2009). Misexpression of those genes in chromosomal females results in testicular development (Koopman et al., 1991; Lambeth et al., 2014).

Estrogen plays a strong role in ovarian differentiation. Modulation of estrogen levels resulted in gonadal feminization in birds, reptiles, and eutherian mammals (Elbrecht and Smith, 1992; Coveney et al., 2001; Milnes et al., 2002; Pieau and Dorizzi, 2004; Lambeth et al., 2013; Guioli et al., 2020). In placental mammals such as mice, estrogen seems to have a role in gonadal maintenance, as estrogen receptor α and β double knock out result in postnatal gonadal sex reversal (Couse et al., 1999). In birds, exogenous modulation of estrogen levels can influence the embryonic gonadal fate, independent of the genetic sex. Injection of fadrozole, an estrogen synthesis inhibitor, in female embryos results in testicular development, upregulating *DMRT1* and *AMH* and downregulating *FOXL2* and *aromatase* (Elbrecht and Smith, 1992; Smith et al., 2003; Hirst et al., 2017; Estermann et al., 2021b). In some cases, these gonads reverted to ovotestis, upregulating aromatase at around day 7–9 of development (Nishikimi et al., 2000; Vaillant et al., 2001a; Estermann et al., 2021b). Our results agree with these reports, showing both aromatase and SOX9 expression in the same gonads at E9.5 and E12.5, upon fadrozole treatment (Figures 1C,D). Surprisingly, aromatase-expressing embryonic pre-granulosa cells were confined to the apical region of the gonad whereas the SOX9 positive Sertoli-like cells were present at the base of the gonad, suggesting that some cell populations are more susceptible to redifferentiation. Two main Sertoli cell types were identified in chicken embryonic testis (Estermann et al., 2020). One population located in the most apical region of the gonad expressing low levels of Sertoli markers *SOX9* and *DMRT1*, and the other located more basally, expressing higher levels of *SOX9* and *DMRT1* (Estermann et al., 2020). *DMRT1* is required to maintain the Sertoli cell identity in testis (Smith et al., 2009; Matson et al., 2011; Ioannidis et al., 2021). Lower *DMRT1* expression in the apical region could explain the higher susceptibility of these supporting cells to transdifferentiate. On the other hand, a higher sensitivity to estrogens could be also responsible to this phenomenon, although this remains unexplored. Further research is required to evaluate why the apical supporting cell population is more sensitive to transdifferentiation than the basal one.

The process of supporting cell re-differentiation coincides with the upregulation of PAX2 in the gonads, a marker normally only expressed in undifferentiated supporting cells (Estermann et al., 2020; Estermann et al., 2021a). This suggests that Sertoli cells could revert into a more undifferentiated or bipotential state before they “re-differentiate” into pre-granulosa cells. These PAX2^+^ “undifferentiated” supporting cells are located between the SOX9^+^ Sertoli-like and the aromatase positive pre-granulosa cells. In transdifferentiating adult mouse gonads, cells expressing both FOXL2 and SOX9 are detected, suggesting the presence of intermediate double positive cell states (Matson et al., 2011). Further research is required to confirm if, in fact, these are three independent populations in the chicken or if double or triple positive cells are detected. PAX2, SOX9 and aromatase or FOXL2 co-localisation in the same gonadal sections will be required to address this question.

It is curious that PAX2 was upregulated only during fadrozole mediated but not in estrogen mediated sex reversal. This could suggest that female to male sex reversal occurs differently than male to female sex reversal, perhaps due to the early elevated expression of DMRT1 in males that prevents such de-differentiation. Another possibility is that we missed the PAX2 expression/undifferentiated window in our time series. In fadrozole mediated sex reversal, PAX2 was differentially expressed at E9.5 but not at E6.5 or E12.5. This suggest that this process occurs only at a short period of time. Only E9.5 gonads were evaluated in 17β-estradiol mediated sex reversal. Further research should expand our analysis to other embryonic stages before and after E9.5 to elucidate if pre-granulosa to Sertoli re-differentiation also involves an undifferentiated PAX2^+^ state. Similarly, intermediate states between E6.5 and E12.5 in fadrozole mediated sex reversal are required to precisely define the timeframe of this dedifferentiation and redifferentiation process. This would expand our knowledge in this poorly studied mechanism.

The chicken model is an ideal system to study genetic, environmental, and hormonal factors that control normal or abnormal gonadal differentiation. Estrogenic mediated sex reversal provides a valuable tool to model and study DSDs, especially partial sex reversion and ovotestis development.

## Data Availability

The original contributions presented in the study are included in the article/Supplementary Material, further inquiries can be directed to the corresponding author.
